# Continuous renal replacement therapy rescues severe haemorrhagic fever with renal syndrome in pregnancy: a case report

**DOI:** 10.1186/s12879-020-05638-8

**Published:** 2020-12-03

**Authors:** Xiang Ying, Xiaoli Lai, Xiaoxiao Jin, Linghong Cai, Xiaotian Li

**Affiliations:** 1 Department of Obstetrics, Taizhou Hospital of Zhejiang Province affiliated to Wenzhou Medical University, Taizhou, 317000 Zhejiang Province People’s Republic of China; 2 Department of Obstetrics, Obstetrics and Gynecology Hospital of Fudan University, Shanghai, 200011 Shanghai Municipality People’s Republic of China

**Keywords:** Haemorrhagic fever with renal syndrome, Pregnancy, Continuous renal replacement therapy, Case report

## Abstract

**Background:**

Haemorrhagic fever with renal syndrome (HFRS) is a natural epidemic disease caused by various types of viruses of the genus *Hantavirus*, which are mainly transmitted by contact with the infected rodents and their droppings. Pregnancy complicated with HFRS is rare; however, adverse maternal and foetal outcomes may be noted. In this report, we describe a case involving a pregnant woman with HFRS who was in a state of multiple organ dysfunction syndrome (MODS) and was successfully treated with continuous renal replacement therapy (CRRT).

**Case presentation:**

A 32-year-old pregnant woman at 29 weeks of gestation was hospitalised for a fever and upper respiratory tract infection due to HFRS in winter. Persistent fever, coagulation disorder, thrombocytopenia, electrolyte imbalance, abnormal liver function, and renal failure were noted during the progression of the disease. The patient was treated with CRRT. She recovered after 21 days, and delivered a live infant by caesarean section at 38 weeks of gestation. Furthermore, obvious abnormalities were not detected during the follow-up of the mother and infant at 42 days, 3 months, 6 months, and 1 year after the delivery.

**Conclusions:**

Early diagnosis, timely application of CRRT, and comprehensive treatment may be essential for the successful treatment of patients with HFRS during pregnancy.

## Background

Haemorrhagic fever with renal syndrome (HFRS) is a natural epidemic disease caused by various species of *Hantavirus*, with rodents as the main source of infection. According to statistical data, the fatality rate associated with HFRS was 2.89% in China from 1950 to 2014 [1]. HFRS develops very rarely during pregnancy in China as well as in other countries. The clinical manifestation of HFRS during pregnancy is atypical, and the mortality rate is high. Herein, we report the successful treatment of a case of severe HFRS complicating pregnancy.

## Case presentation

**Fig. 1:**
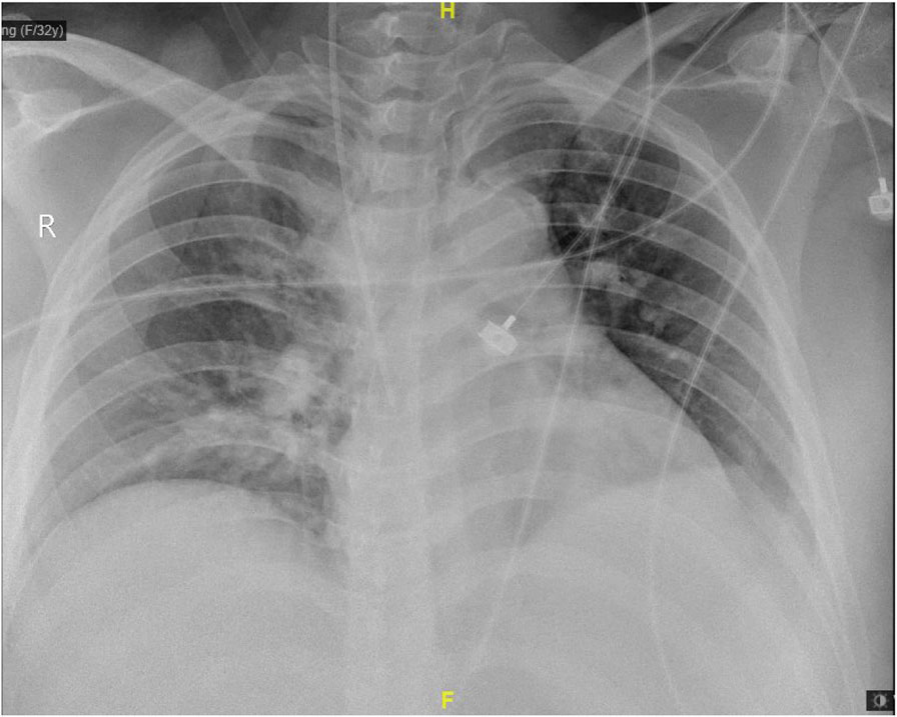
The chest X-ray of the patient when she was on admission

A 32-year-old female (gravida 5, para 1) was referred to our hospital on January 23, 2019, at 29 weeks of gestation, because of 10 days’ cough and 3 days’ fever. The patient had undergone a caesarean section in 2007. The patient was healthy prior to this episode, and she had no significant family history. During the current pregnancy, an oral glucose tolerance test (OGTT) performed at 24 weeks of gestation indicated abnormal glucose tolerance. Insulin aspart was administered to reduce the blood sugar levels, and her blood glucose level was well controlled with this treatment. On January 13, 2019, the patient developed a cough and visited the local hospital when the cough worsened 3 days later. She was administered “Shuanghuanglian,” a traditional Chinese medicine composed of honeysuckle, scutellaria, and forsythia used for treating colds; however, her symptoms were not relieved by this treatment. Three days before admission, the patient developed a fever of up to 39.8 °C with a worsening cough and chest tightness. Ibuprofen and penicillin were administered before admission, but the symptoms did not improve. On admission, the patient appeared to be in pain, was lethargic, and had shortness of breath. The foetal heart rate was 145–190 beats per minute and B ultrasound showed that development of the foetus was consistent with gestational age. The patient’s temperature was 37.9 °C; pulse rate, 155 beats per minute; respiratory rate, 22 times per minute; and blood pressure, 91/67 mmHg. Blood gas analysis showed metabolic acidosis with respiratory alkalosis, as follows: pH, 7.46[Reference ranges 7.35–7.45]; PaO_2_, 70 mmHg [Reference ranges 83-108 mmHg]; base excess, − 6.4 mmol/L [Reference ranges − 3.0 − + 3.0 mmol/L]; and PaCO_2_, 23 mmHg [Reference ranges 35-45 mmHg]. Other laboratory examinations indicated thrombocytopenia (haemoglobin [Hb] level, 140 g/L [Reference ranges 115–150 g/L]; platelet [PLT] count, 40*10^9/L)[Reference ranges 125–350*10^9/L], inflammatory reaction (white blood cell [WBC] count, 12.1*10^9/L[Reference ranges 3.5–9.5*10^9/L]; and C-reactive protein [CRP] level, 120.1 mg/L) [Reference range < 8 mg/L], coagulation disorders (activated partial thromboplastin time [APTT], 56.4 s [Reference ranges 28.00–42.00 s]; prothrombin time [PT], 31.9 s [Reference ranges 11.00–14.50 s]; fibrinogen [FIB] level, 4.44 g/L [Reference ranges 2.00–4.00 g/L]), liver dysfunction (alanine aminotransferase [ALT] level, 39 U/L [Reference ranges 7.00–40.00 U/L]; aspartate aminotransferase [AST] level, 152 U/L [Reference ranges 13.00–35.00 U/L]; and serum albumin level, 28.5 g/L [Reference ranges 40.00–55.00 g/L]), and renal damage (serum creatinine level, 248 μmmol/L [Reference ranges 46.00–92.00μmmol/L]; serum urea level, 6.81 mmol/L [Reference ranges 2.50–6.10 mmol/L]; urine occult blood,**-**; urine protein,+++). The chest radiograph (Fig. 1) showed a patch shadow in the right lung, which indicated pneumonia. However, the patient tested negative using ELISA for immunoglobulin M (IgM) for respiratory viral antigens, respiratory syncytial virus antigens, adenovirus, influenza A and B and parainfluenza viral antigens, and Mycoplasma pneumonia-IgM. The sputum, blood, and urine cultures were all found to be normal. Moreover, the patient was a farmer in the countryside in the Zhejiang province in China, where mice may be present in the living environment. The patient tested positive for HFRS-IgM detecting by ELISA. Thus, the aforementioned clinical manifestations and laboratory test results showed that the patient had severe HFRS, pneumonia (pathogen unknown), severe sepsis, septic shock, and MODS, including acute respiratory failure, abnormal liver function, acute kidney injury (AKI), and disseminated intravascular coagulation (DIC).

After admission, deep venepuncture was used to monitor the central venous pressure of the patient, and an indwelling pulse contour cardiac output (PICCO) catheter was used to monitor the cardiac output and maintain haemodynamic stability. The main treatment modalities included continuous high-flow mask oxygen (5 L/min), intravenous imipenem (500 mg every 6 h), oral oseltamivir phosphate, liver protection (Reduced glutathione), and blood sugar regulation. The urine output was only 150 ml, 16.7 h after admission. On the first day of admission, the laboratory examination results revealed that the patient had thrombocytopenia (25 × 10^9^/L), deterioration in coagulation function (APTT, 62.8 s and PT, 36.8 s), and renal dysfunction (creatinine level, 250 μmol/L and urea 7.0 mmol/L). On day 2, continuous veno–venous hemodiafiltration (CVVHDF) was administered using a hemofiltration replacement basic solution (Qingshan Likang Co, Chengdu, China), 0–20 ml of 10% KCl (adjusted according to the serum potassium levels), and 5% sodium bicarbonate with micropump maintenance of 93 ml/h. The blood flow rate was set at 200 ml/min; the replacement fluid volume was 1500 ml/min, and the provisional dehydration rate was 300 ml/h. The haemofilter was flushed with 100 ml normal saline at hourly intervals. We monitored the results of the blood gas analysis, hemagglutination, and electrolytes levels and adjusted the programme, as necessary. In addition, the patient received treatments aimed at targeting the infection (500 mg imipenem-cilastatin sodium administered intravenously every 6 h) and virus (oseltamivir) while providing symptomatic supportive measures (blood transfusion, albumin supplementation, blood glucose control, and liver protection). On day 6, the patient’s 24-h urine volume was 80 ml, her body temperature was normal, and her vital signs were stable. The laboratory tests showed that the inflammatory index had improved. We changed the antibiotics to intravenous administration of 4.5 g piperacillin/tazobactam every 8 h. On day 9, the urine volume returned to normal (700 ml/24 h). On day 10, haemodialysis was discontinued, and furosemide was administered to control the fluid output. Other medications were continued. On day 13, the patient entered the polyuric phase (urine volume 4900 ml/24 h), and the patient was weakly positive for HFRS-IgM. On day 20, the 24-h urine volume was 2600 ml. The patient was discharged on day 21 with outpatient follow-up. Table 1 shows a timeline of the laboratory assessment for the patient. Finally, the patient gave birth to a live baby boy by caesarean section at 38 weeks of gestation in our hospital and Apgar score of the new-born was 10. Because HFRS-IgG can’t be tested in our hospital, the Hantavirus Hantaan IgG enzyme-linked immune sorbent assay kit (IBL International GmbH, Hamburg, Germany) was used to detect IgG antibodies against the serotype Hantaan of Hantavirus, according to the manufacturer’s protocol. At the time of delivery, the HFRS-IgM and HFRS-IgG levels in the umbilical cord were assessed; the umbilical cord samples tested negative for HFRS- IgM and positive for HFRS-IgG. The neonatal serum samples still tested negative for HFRS-IgM and positive for HFRS-IgG 2 days after birth. Obvious abnormalities were not detected during the follow-up of the mother or infant at 42 days, 3 months, 6 months and 1 year after the delivery. Besides, the infant tested negative for HFRS-IgM and HFRS-IgG at 6 months after the delivery. The mother tested positive for HFRS-IgG and the infant tested negative for HFRS- IgG at 1 year after the delivery.

**Table 1:** Clinical laboratory data of the pregnant women with EHF

Index	WBC (10^9/L)	Hb (g/L)	PLT (10^9/L)	APTT (seconds)	PT (seconds)	Urine occult blood	Urine protein	AST (U/L)	Serum albumin (g/L)	Serum creatinine (μmmol/L)	Serum urea (mmol/L)
Date											
1.25	17.2	104	43	60.4	22.1	3+	3+	80	21	208	5.82
1.28	10.7	85	31	47.2	15.9			29	26.3	319	5.95
1.31	6.3	75	75	43.4	4.46			18	27.8	360	7.29
2.6	7.0	73	133	41.3	17.6			16	32.9	272	9.31
2.12	5.6	85	142					16	36.6	104	2.36
Reference ranges	3.50–9.50	115.00–150.00	125.00–350.00	28.00–42.00	11.00–14.50	negative	negative	13.00–35.00	40.00–55.00	46.00–92.00	2.50–6.10

## Discussion and conclusions

This pregnant woman presented with a “fever and cough” in the early stages, and was misdiagnosed with a lower respiratory tract infection. HFRS was not considered until she developed septic shock and multiple organ failure. Comprehensive treatment including CRRT was administered immediately after a definite diagnosis was made, and the mother and foetus recovered with a shorter disease course than that reported previously [2].

The maternal coagulation factors and placental synthesis of thrombogenic substances increase during pregnancy. HFRS during pregnancy may lead to severe complications, such as DIC. The mortality rate associated with pregnant HFRS patients is higher than that associated with non-pregnant patients [2]. Moreover, the HFRS virus can be transmitted vertically through the placenta, which leads to adverse perinatal outcomes such as abortion, premature delivery, stillbirth, and foetal malformation. Figurnov [3] reported a case of hydrocephalus in the offspring of a pregnant woman who was infected with *Hantavirus* at 31 weeks of gestation. In this case, the pregnant woman was infected with *Hantavirus* during the third trimester (29 weeks of gestation). We found that the umbilical cord samples and the serum samples of the neonate tested negative for HFRS-IgM and positive for HFRS-IgG, indicating that maternal Hantavirus IgG can be transferred to the foetus via the placenta without IgM synthesis. Moreover, HFRS-IgM and HFRS-IgG levels were undetectable in the infant 6 months after the delivery, suggesting that IgG specific antibody provided by the mother may protect the foetus from viral infection. The mother still tested positive for HFRS-IgG while the infant tested negative for HFRS-IgG at 1 year after the delivery. Thus, these findings indicate that Hantavirus may not infect the foetus through the placenta. However, there are some limitations of this report. It would be better if efforts were made to detect Hantavirus RNA in the umbilical cord and serum of the infant. The patient breastfed the baby postpartum and obvious abnormalities were not detected during the follow-up conducted at 42 days, 3 months, 6 months, and 1 year after the delivery, implying that HFRS may not have adverse effects on the offspring of this patient in the near future.

This case could have been easily misdiagnosed. On one hand, HFRS cases are mainly concentrated in the Northern and North-eastern parts of China during the autumn season. Our patient developed HFRS during the non-epidemic period and resided in an area not prone to the HFRS epidemic; moreover, the clinical manifestation was not typical. Thus, it would be unlikely for an obstetrician to consider the diagnosis of HFRS during the first visit. After obtaining a complete medical history, we found that the patient was a farmer in the countryside in the Zhejiang province in China, where mice may be present in the living environment. The patient was further tested for HFRS-IgM. Thus, the complete medical history of patients who present with a fever must be obtained. On the other hand, the clinical manifestations of HFRS in pregnant women are non-specific and should be distinguished from those of pregnancy-related diseases such as HELLP syndrome, acute fatty liver, acute hepatitis, or haemolytic uremic syndrome-thrombotic thrombocytopenic purpura (HUS-TTP). HELLP is a severe complication of a hypertensive disorder complicating pregnancy, mainly manifesting with an abnormal liver function, thrombocytopenia, and haemolysis, with renal function damage in some patients. However, it generally does not manifest as a fever. Acute fatty liver in pregnancy is a serious condition and manifests as digestive tract symptoms, jaundice, and hepatic encephalopathy. Acute renal failure may also develop, resulting in oliguria, shock and other detrimental sequelae. Fever, infection, and other symptoms are not detected commonly. Pregnancy complicated with acute severe viral hepatitis presents with an increase in the bilirubin levels, decreased serum cholinesterase levels, and elevated blood ammonia levels, and it can be identified by positivity for hepatitis viral markers. The clinical manifestations of HUS-TTP are microvascular haemolytic anaemia, progressive thrombocytopenia, acute renal failure, fever, and nervous system symptoms, which can easily lead to a misdiagnosis. Positivity for HFRS- IgM can aid in differentiating between HFRS and all the aforementioned diseases.

Disease severity and the effects of therapy on the mother and foetus should be considered in the treatment of HFRS during pregnancy. The United States Food and Drug Administration (FDA) states that renal replacement therapy (RRT) is the only treatment for AKI. However, a few studies have reported on the administration of RRT in pregnant women. The main RRT modalities are intermittent haemodialysis (IHD), peritoneal dialysis (PD), and CRRT. With improvements in RRT, the application of CRRT for the treatment of HFRS and AKI is gradually increasing [4]. There is no statistical difference between CRRT and IHD with respect to mortality and renal function recovery [5]; however, better hemodynamic stability and fluid balance can be achieved with CRRT. Thus, it has become the first-line treatment during AKI replacement therapy in pregnancy. CVVHDF is a combination of continuous veno–venous haemofiltration (CVVH) and continuous veno-venous haemodialysis (CVVHD). CVVHDF can quickly remove the blood urea nitrogen, creatinine, and inflammatory mediators during the treatment of AKI and has been gradually used for the treatment of HFRS patients [6]. Severe AKI and MODS developed in our patient, and she was in a state of haemodynamic instability. Prompt administration of CRRT (CVVHDF) resulted in positive maternal and foetal outcomes. Thus, CRRT treatment can improve the renal function and intrauterine environment of pregnant women with HFRS, increasing the incidence of successful outcomes of pregnancy. However, the optimal timing and indication for CRRT administration in pregnant women should be explored. The timing of pregnancy termination depends on the progression of the disease, gestational age and foetal condition. MODS is not an absolute indication for immediate termination of pregnancy. With proper management of the disease, the patient can continue the pregnancy. Termination of pregnancy is recommended when the disease develops quickly. Therefore, evaluating the condition of the mother and the foetus is important for determining whether pregnancy should be continued. Furthermore, the family member’s wishes should also be considered. Moreover, a multidisciplinary team, including anaesthesiologists, obstetricians, infectious disease specialists, and intensivists, is required to determine the most appropriate treatment and improve perinatal outcomes.

## Data Availability

The datasets analysed during the current study are not publicly available due to patient privacy concerns but are available from the corresponding author on reasonable request.

## Abbreviations

AKI: Acute kidney injury
WBC: White blood cell
Hb: Haemoglobin
PLT: Platelets
CRP: C-reactive protein
APTT: Activated partial thromboplastin time
PT: Prothrombin time
FIB: Fibrinogen
ALT: Alanine aminotransferase
AST: Aspartate aminotransferase
CRRT: Continuous renal replacement therapy
CVVH: Continuous veno-venous hemofiltration
CVVHD: Continuous veno-venous haemodialysis
CVVHDF: Continuous veno-venous haemofiltration
DIC: Disseminated intravascular coagulation
FDA: Food and Drug Administration
HFRS: Haemorrhagic fever with renal syndrome
HUS-TTP: Haemolytic uremic syndrome-thrombotic thrombocytopenic purpura
IHD: Intermittent haemodialysis
MODS: Multiple organ dysfunction syndrome
OGTT: Oral glucose tolerance test
PD: Peritoneal dialysis
PICCO: Indwelling pulse contour cardiac output catheter
